# Inducible modulation of miR-204 levels in a zebrafish melanoma model

**DOI:** 10.1242/bio.053785

**Published:** 2020-11-06

**Authors:** Samanta Sarti, Raffaella De Paolo, Chiara Ippolito, Angela Pucci, Letizia Pitto, Laura Poliseno

**Affiliations:** 1 Oncogenomics Unit, CRL-ISPRO, Pisa 56124, Italy; 2 Institute of Clinical Physiology, CNR, Pisa 56124, Italy; 3 University of Siena, Siena 53100, Italy; 4 Unit of Histology and Human Embryology, Department of Clinical and Experimental Medicine, University of Pisa, Pisa 56126, Italy; 5 Histopathology Department, Pisa University Hospital, Pisa 56126, Italy

**Keywords:** Zebrafish, Melanoma model, miniCoopR-I, *miR-204*, Pigmentation

## Abstract

Here, we present miniCoopR-I, an inducible upgrade of the constitutive miniCoopR vector. We developed miniCoopR-I-sponge-204 and miniCoopR-I-pre-miR-204 vectors and we successfully tested them for their ability to achieve time- (embryo/juvenile/adult) and space- (melanocytic lineage) restricted inhibition/overexpression of *miR-204*, a positive modulator of pigmentation previously discovered by us. Furthermore, melanoma-free survival curves performed on induced fish at the adult stage indicate that *miR-204* overexpression accelerates the development of BRAFV600E-driven melanoma. miniCoopR-I allows study of the impact that coding and non-coding modulators of pigmentation exert on melanomagenesis in adult zebrafish, uncoupling it from the impact that they exert on melanogenesis during embryonic development.

This article has an associated First Person interview with the first author of the paper.

## Abstract (precis)

**Summary:** Sarti et al. describe miniCoopR-I inducible vectors, a refined tool to study the impact exerted by pigmentation on melanogenesis (during embryonic development) versus melanomagenesis (in adult zebrafish).

## INTRODUCTION

In humans, melanotic melanomas are characterized by worse prognosis (Cancer Genome Atlas Network, 2015) and higher aggressiveness compared to amelanotic ones (Kim et al., 2017; Damsky et al., 2011). Furthermore, they display higher resistance to radio- (Brożyna et al., 2016), chemo- (Xie et al., 2009; Huang et al., 2012; Chen et al., 2006; Sanchez-del-Campo et al., 2009), photodynamic- (Sharma and Davids, 2012) and, as we discovered, targeted therapy (Vitiello et al., 2017). The *hsa-miR-204* family is composed of *hsa-miR-204-5p* (*hsa-miR-204*) and *hsa-miR-211-5p* (*hsa-miR-211*), whose genes are located in intron 6 of *TRPM3* and *TRPM1*, respectively. TRPM3 voltage-gated ion channel is expressed in various brain districts and in the retina (https://www.proteinatlas.org/ENSG00000083067-TRPM3/tissue). TRPM1 voltage-gated ion channel is expressed in the retina (mutations are associated with congenital stationary night blindness (AlTalbishi et al., 2019) and in melanocytes (https://www.proteinatlas.org/ENSG00000134160-TRPM1), where it is transcriptionally regulated by and acts as a downstream effector of MITF (Vitiello et al., 2017).

In human melanoma cells, both microRNAs are negatively regulated by BRAFV600E through the ERK pathway, but they exert different functions. *hsa-miR-204*, which is under the transcriptional control of STAT3, mediates the anti-motility activity of the BRAFV600E inhibitor vemurafenib. Conversely, *hsa-miR-211* mediates the pro-pigmentation activity of vemurafenib. In turn, pigmentation is an adaptive cellular response that limits the efficacy of vemurafenib itself: *hsa-miR-211* overexpression makes melanoma cells less sensitive to vemurafenib, and this is reverted by treatment with phenylthiourea (an inhibitor of melanin biosynthesis), demonstrating a dependency on *miR-211* pro-pigmentation activity. Conversely, vemurafenib efficacy is increased by the concomitant inhibition of the miRNA by means of the LNA-211 inhibitor (Vitiello et al., 2017; Vitiello et al., 2018).

According to miRBase (http://mirbase.org), in zebrafish there is no *miR-211*. However, *dre-miR-204-5p* (*dre-miR-204*) is present, has the same sequence as human *miR-204* and derives from three gene copies: *dre-miR-204-1* on chromosome 5, *dre-miR-204-2* on chromosome 7 and *dre-miR-204-3* on chromosome 25. *dre-miR-204-3* is poorly characterized, but *dre*-*miR-204-1* and *dre-miR-204-2* are known to be located in an intron of *trpm3* and *trpm1a,* respectively (https://www.ensembl.org/index.html). During development of the embryo, *trpm3* is expressed in neural crest cells and then in the ocular lens, as well as in different areas of the brain (Kastenhuber et al., 2013). *trpm1a* is also expressed in neural crest cells, but later on its expression becomes restricted to retinal pigment epithelium and melanocytes (Kastenhuber et al., 2013), where it falls under the transcriptional control of Mitfa (Seberg et al., 2017).

In the absence of experimental data about the involvement of *dre-miR-204* in melanoma, we considered the similarities in expression patterns and transcriptional regulation as an indication that *dre-miR-204* covers for *hsa-miR-211* functions. Therefore, in order to confirm the impact exerted by pigmentation on BRAFV600E-driven melanoma *in vivo*, we decided to take advantage of the melanoma-prone *Tg(mitfa:BRAFV600E);p53−/−;mitfa−/−* transgenic line and to modulate the levels of *miR-204* in zebrafish, by means of the miniCoopR vector (Ceol et al., 2011). This vector contains a *mitfa* minigene (*mitfa* promoter, ORF and 3′UTR) that allows retrieval of the expression of the transcription factor. In this way, the development of melanocytes is triggered and, in presence of mutant BRAFV600E and in the absence of *p53*, melanoma formation is allowed. miniCoopR vector also contains a *mitfa* promoter-driven expression cassette that allows restriction of the expression of a gene of interest into melanocytes. In turn, this means that melanocytes express, simultaneously, the gene of interest and BRAFV600E. Therefore, miniCoopR allows us to establish whether the gene of interest affects BRAFV600E-induced melanomagenesis.

In order to restrict the modulation of *miR-204* levels to the adult stage, avoiding the consequences it has on the embryonic development of the melanocytic lineage, we upgraded the miniCoopR vector into the inducible miniCoopR-I, as we describe below.

## RESULTS AND DISCUSSION

### Constitutive modulation of *miR-204* expression levels affects melanocyte content in zebrafish embryos of the *Tg(mitfa:BRAFV600E);p53−/−;mitfa−/−* line

**Fig. 1.**
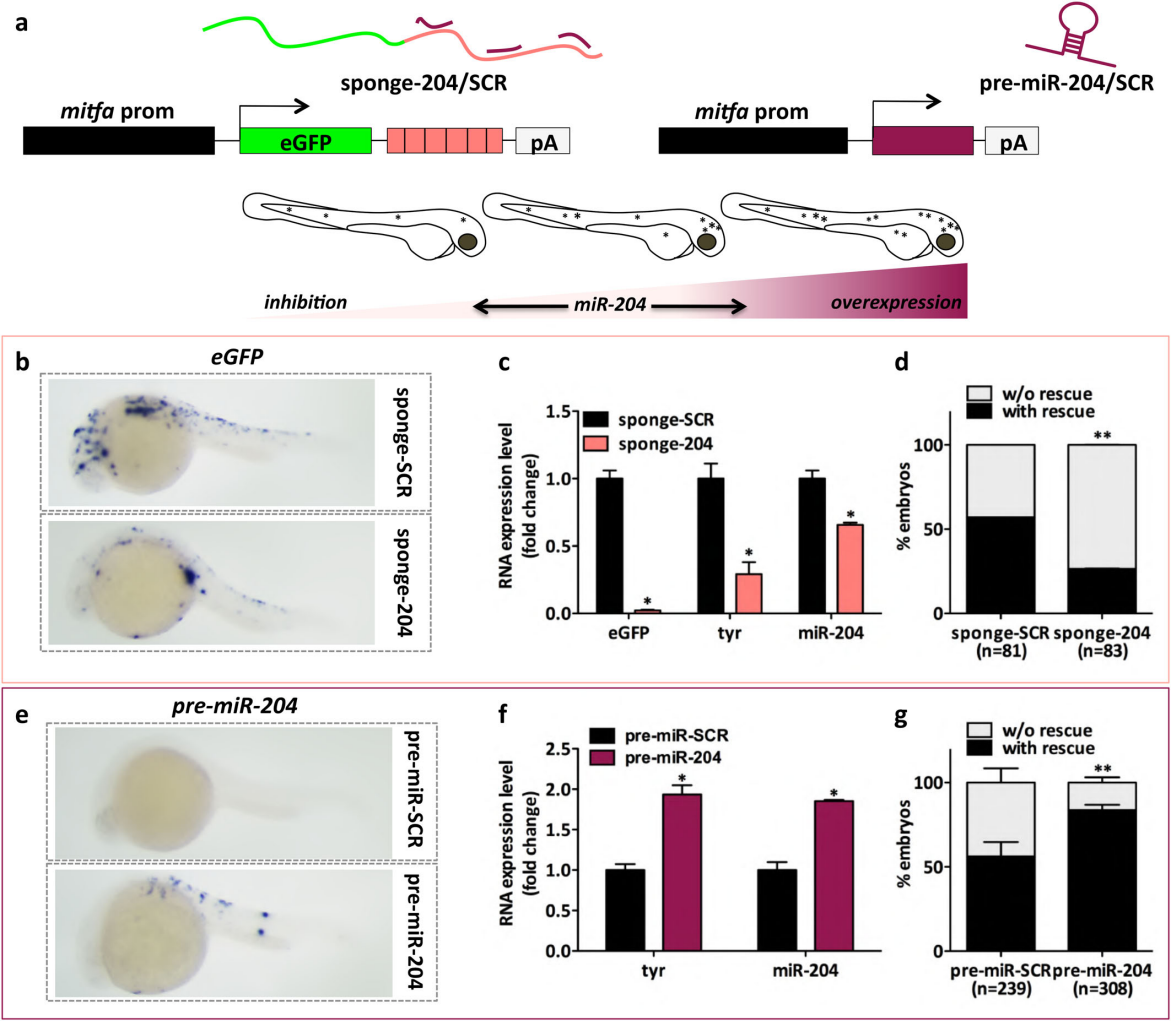
The constitutive modulation of *miR-204* expression levels affects melanocyte content in embryos of the *Tg(mitfa:BRAFV600E);p53−/−;mitfa−/−* line. (A) (upper) Schematic representation of the miniCoopR vectors used for the inhibition and the overexpression of *miR-204*. On the left, *miR-204* inhibition is achieved using a sponge construct that contains six imperfect binding sites for *miR-204* downstream of eGFP coding sequence (miniCoopR-sponge-204). On the right, *hsa-pre-miR-204* precursor is used to achieve the overexpression of mature *miR-204* (miniCoopR-pre-miR-204). As negative controls, miniCoopR-sponge-SCR and miniCoopR-pre-miR-SCR are used, respectively. (lower) Cartoon summarizing the effects observed on embryos’ pigmentation. (B) *eGFP* mRNA detected by ISH 24 h after the injection of miniCoopR-sponge-SCR or miniCoopR-sponge-204 in 1-cell embryos. (C) *eGFP*, *tyr* and mature *miR-204* levels measured by qRT-PCR at 7 dpf, upon the injection of miniCoopR-sponge-SCR or miniCoopR-sponge-204 in 1-cell embryos. (D) Percentage of 7dpf embryos showing rescued melanocytes, upon the injection of miniCoopR-sponge-SCR or miniCoopR-sponge-204 at the 1-cell stage. (E) *pre-miR-204* RNA detected by ISH 24 h after the injection of miniCoopR-pre-miR-SCR or miniCoopR-pre-miR-204 in 1-cell embryos. (F) *tyr* and mature *miR-204* levels measured by qRT-PCR at 7 dpf, upon the injection of miniCoopR-pre-miR-SCR or miniCoopR-pre-miR-204 in 1-cell embryos. (G) Percentage of 7 dpf embryos showing rescued melanocytes, upon the injection of miniCoopR-pre-miR-SCR or miniCoopR-pre-miR-204 at the 1-cell stage. Statistically significant differences are indicated with asterisks: *P<0.05, **P<0.01.

Once double checked that endogenous *miR-204* levels are not altered in *Tg(mitfa:BRAFV600E);p53−/−;mitfa−/−* embryos compared to wild-type embryos (data not shown), to achieve the inhibition of mature *miR-204*, irrespectively from the gene copy it is transcribed from, we created a sponge construct in which six imperfect binding sites for the microRNA are located downstream of the eGFP coding sequence, so that a 3′UTR is mimicked (miniCoopR-sponge-204). Conversely, to achieve the overexpression of mature *miR-204*, we used *hsa-pre-miR-204* precursor (miniCoopR-pre-miR-204) (Fig. 1A).

miniCoopR-sponge-204 or miniCoopR-pre-miR-204 and relative controls (miniCoopR-sponge-SCR or miniCoopR-pre-miR-SCR, respectively) were injected into 1-cell embryos of the *Tg(mitfa:BRAFV600E);p53−/−;mitfa−/−* line. ISH analysis of *eGFP* mRNA indicated that the sponge constructs are already expressed at 24 hpf, while the lower signal observed in embryos injected with sponge-204 compared to sponge-SCR is consistent with reporter destabilization due to microRNA binding (Lai et al., 2018) (Fig. 1B). In addition, while confirming the decrease in *eGFP* mRNA levels, qRT-PCR analysis performed at 7 dpf allowed us to demonstrate that sponge-204 causes the expected decrease in the levels of *miR-204* (Fig. 1C) (Lai et al., 2018; Yin et al., 2012). Finally, we observed that such a decrease is accompanied by a decrease in the levels of the melanocyte marker *tyr* (Fig. 1C) and in the percentage of embryos showing melanocytes rescue (Fig. 1D). Conversely, *miR-204* overexpression led to opposite results: increases in *(pre)-miR-204* levels (Fig. 1E,F), *tyr* levels (Fig. 1F) and percentage of rescued embryos (Fig. 1G).

We injected the sponge and pre-miR constructs in 1-cell embryos of the *p53−/−;mitfa−/−* transgenic zebrafish line as well, and the results we obtained were comparable to those obtained in the *Tg(mitfa:BRAFV600E);p53−/−;mitfa-/-* line (Fig. S1). Furthermore, 48/72/96 hpf embryos of the *Tg(mitfa:BRAFV600E);p53−/−;mitfa−/−* line previously injected with miniCoopR-sponge-SCR/204 or miniCoopR-pre-miR-SCR/204 were subjected to qRT-PCR and ISH detection of markers of neural crest differentiation into glia cells and melanocytes. As summarized in Fig. S2a, these markers are *sox10* for bipotent stem cells (yellow), *gfap*, *plp1* and *s100b* for glia cells (grey), and *mitfa*, *dct* and *tyr* for committed-differentiated melanocytes (brown). *miR-204* inhibition did not result in any change in markers expression (Fig. S2B,C, upper panels, D, upper panel). However, upon *miR-204* overexpression an increase in *sox10* levels was observed in embryos at 96 hpf (Fig. S2B, lower panel). Such an increase was not accompanied by any change in the expression levels of glia and melanocyte markers (Fig. S2C, lower panels, d, lower panel), but is still consistent with the enhanced melanocyte rescue observed at a later time point (7 dpf, Fig. 1G).

Overall, the data described above indicate that, during embryonic development, *miR-204* impinges on neural crest lineage and favors melanocyte differentiation independently of BRAFV600E. Therefore, they confirm that *miR-204* does play a role in the melanocytic lineage in zebrafish (as *miR-211* does in humans). On the other side they also suggest that, in order to study the effects exerted by *miR-204* on melanomagenesis, it is necessary to restrict its expression to the adult stage, bypassing embryonic development and hence avoiding the misleading factor that would be represented by an uneven melanocyte number.

### miniCoopR-I vectors for the inducible expression of pigmentation modulators in adult zebrafish

**Fig. 2.**
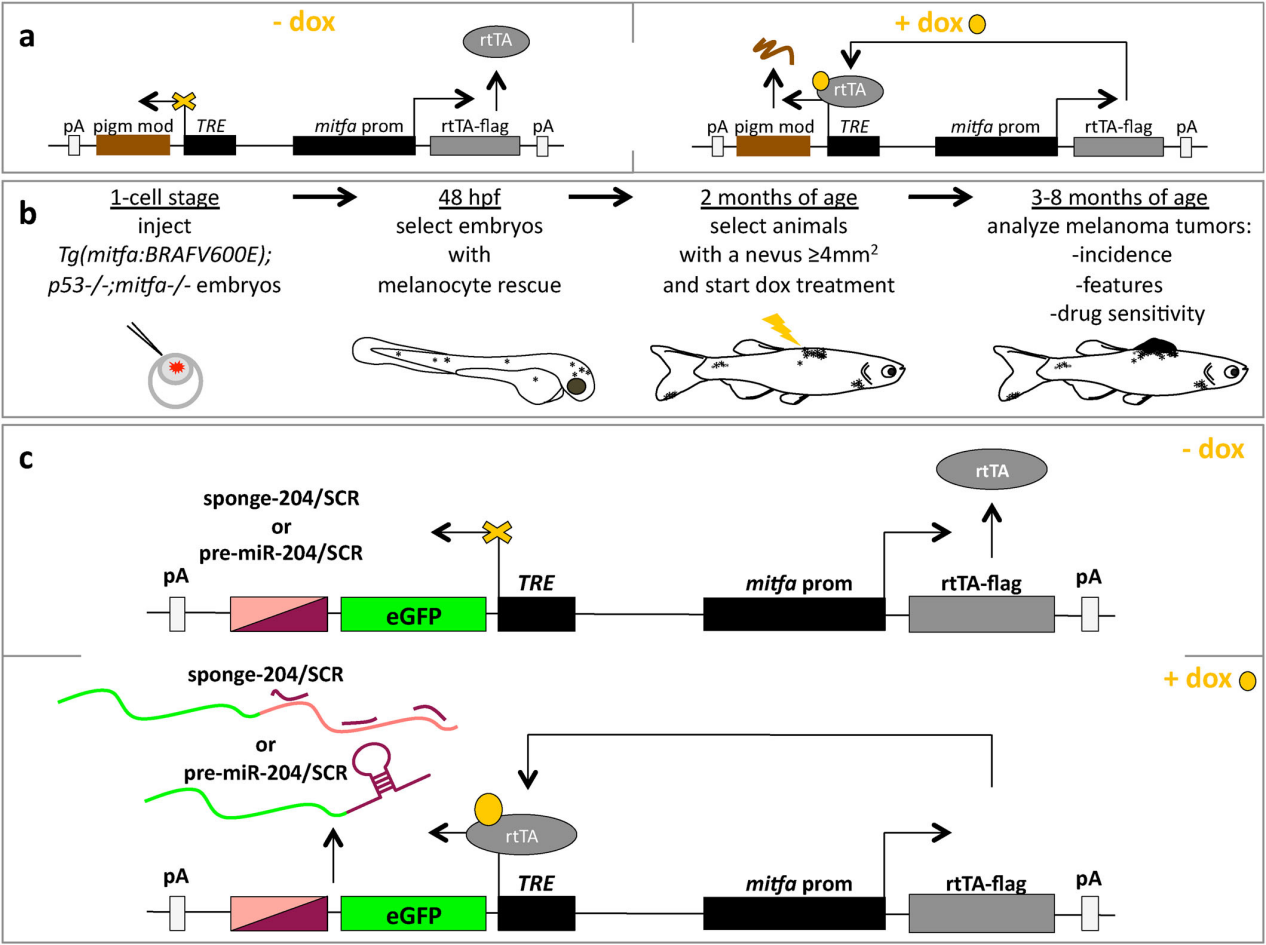
**Description of miniCoopR-I vectors for the doxycycline-inducible overexpression of negative and positive modulators of pigmentation.** (A) Cartoon describing the general functioning of miniCoopR-I vector. *mitfa* promoter drives the expression of the reverse tetracycline-controlled transactivator (rtTA, grey). The gene of interest (a pigmentation modulator, brown) is located downstream of tetracycline responsive element (TRE) and gets expressed only when doxycycline (dox, yellow) is added to fish water, so that it can bind to and activate the rtTA. (B) Schematic representation of the experimental protocol to be followed to study the pigmentation modulator of interest. 20–30 pg of miniCoopR-I vector are injected together with 20–30 pg of Tol2 transposase mRNA in 1-cell stage *Tg(mitfa:BRAFV600E);p53;mitfa−/−* embryos. In line with the protocol described in (Ceol et al., 2011; Iyengar et al., 2012; Painter and Ceol, 2014), successfully injected embryos are selected at 48 hpf on the basis of the presence of rescued melanocytes. Then, at 2 months of age, they are selected on the basis of the presence of nevi ≥4 mm^2^. At this time point, selected fish are treated with dox (10 uM in fish water), irrespectively of sex. At 3–8 months of age, the indicated parameters are analyzed with the appropriate techniques. (C) Cartoon describing the general functioning of miniCoopR-I-sponge-204/SCR (*miR-204* inhibition) and of miniCoopR-pre-miR-204/SCR (*miR-204* overexpression).

**Fig. 3.**
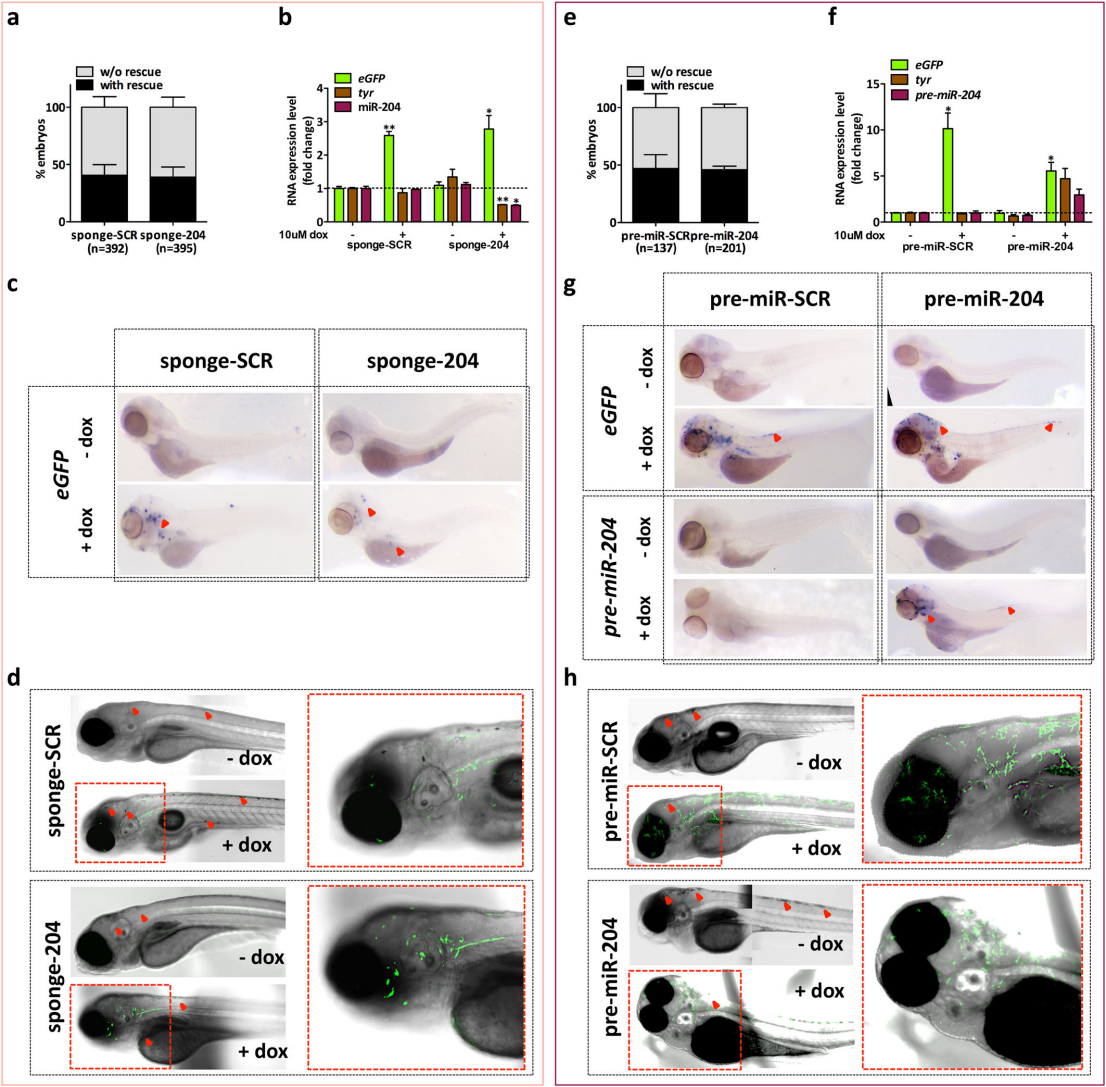
**Inducible modulation of *miR-204* expression levels in embryos of the *Tg(mitfa:BRAFV600E);p53−/−;mitfa−/−* line.** (A) Embryos injected with miniCoopR-I-sponge-SCR or miniCoopR-I-sponge-204 were observed at 7 dpf. The percentage of embryos with and without melanocyte rescue does not differ significantly between the two experimental groups. (B–D) 1-cell embryos injected with miniCoopR-I-sponge-SCR or miniCoopR-I-sponge-204 were selected at 48 hpf for melanocyte rescue and treated with 10 uM dox for 2 days, then the levels of *eGFP*, *tyr* and mature *miR-204* were measured by qRT-PCR (B), *eGFP* mRNA was detected by ISH (C) and eGFP fluorescent protein was detected by confocal microscopy (D). (E) Embryos injected with miniCoopR-I-pre-miR-SCR or miniCoopR-I-pre-miR-204 were observed at 7 dpf. The percentage of embryos with and without melanocyte rescue does not differ significantly between the two experimental groups. (F–H) 1-cell embryos injected with miniCoopR-I-pre-miR-SCR or miniCoopR-I-pre-miR-204 were selected at 48 hpf for melanocyte rescue and treated with 10 uM dox for 2 days, then the levels of *eGFP*, *tyr* and *pre-miR-204* were measured by qRT-PCR (F), *eGFP* and *pre-miR-204* RNAs were detected by ISH (G) and eGFP fluorescent protein was detected by confocal microscopy (H). Red arrows indicate rescued melanocytes, which are visible independently from dox treatment, * indicates statistically significant differences, *P<0.05, **P<0.01.

**Fig. 4.**
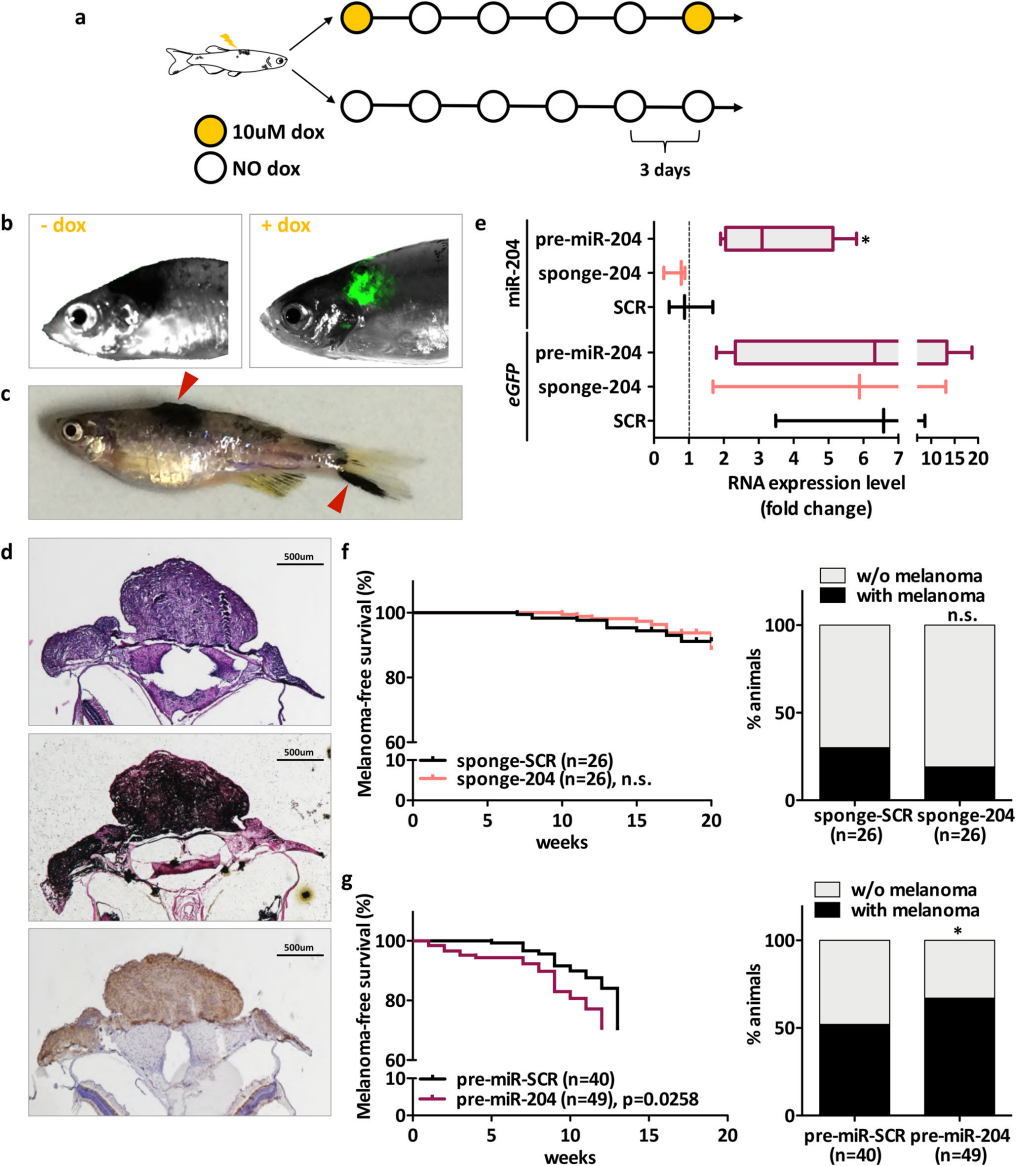
**Effect of *miR-204* modulation on melanoma incidence.** (A–D) Characterization of melanoma tumors that develop in adult fish of the *Tg(mitfa:BRAFV600E);p53-/-;mitfa-/-* line, injected with miniCoopR-I vectors at 1-cell stage. (A) Schematic representation of the dox-treatment schedule used. (B) eGFP fluorescence is detectable only upon treatment with dox. eGFP fluorescence was detectable in two out of three fish tested, one of which is shown in the pictures. (C) Macroscopic appearance of a fish with melanomas (red arrows). (D) Microscopic features of a melanoma tumor that developed on the head and was excised 1 month after it became visible: H&E (upper), Fontana Masson (middle), anti-BRAFV600E (lower). Images were acquired with a 4x objective. Scale bar: 500um. (E) qRT-PCR quantification of mature *miR-204* levels. 1 month after they became visible, tumors that developed on the tail were excised from *n*=3 fish injected with SCR vectors, *n*=3 fish injected with miniCoopR-I-sponge-204 vector and *n*=6 fish injected with miniCoopR-I-pre-miR-204 vector. In all these tumors, effective induction of the transgenic construct was confirmed as higher *eGFP* mRNA levels compared to a piece of tail that was collected from a non-induced fish and taken as baseline. (F) Melanoma-free survival curve (left) and percentage of animals with/without melanoma (right) upon injection of miniCooR-I-sponge-SCR and miniCoopR-I-sponge-204. (G) Melanoma-free survival curve (left) and percentage of animals with/without melanoma (right) upon injection of miniCooR-I-pre-miR-SCR and miniCoopR-I-pre-miR-204. In melanoma-free survival curves, week count started when the first pulse of 10 uM dox was administered, i.e. at 2 months of age. Statistically significant differences are indicated with asterisks: *P<0.05.

To study the effects that the pigmentation modulator *miR-204* exerts on melanomagenesis in adult fish, while uncoupling the effects it exerts on melanocyte development during embryogenesis, we created an inducible version of the miniCoopR vector. Specifically, inspired by the work of Campbell and colleagues who created a Tol2 gateway-based Tet-ON system (Campbell et al., 2012), we developed miniCoopR-I. This vector allows not only a spatial but also a temporal restriction of the expression of the gene of interest (in this case, a pigmentation modulator). This is because, besides the *mitfa* minigene, the miniCoopR-I vector contains two independent expression cassettes, oriented in opposite directions (Fig. 2A). One cassette restricts the expression of the reverse tetracycline-controlled transcriptional trans-activator (rtTA) into melanocytes by means of the *mitfa* promoter. The other cassette allows the expression of the pigmentation modulator of interest only upon doxycycline (dox) treatment, i.e. when the rtTA becomes able to bind to the tetracycline response element (TRE) promoter. In Fig. 2B, we describe the protocol to be adopted in order to study the impact exerted by the pigmentation modulator on BRAFV600E-driven melanomagenesis in adult fish: *Tg(mitfa:BRAFV600E);p53−/−;mitfa−/−* embryos were injected with the appropriate miniCoopR-I vector at the one cell stage and then selected for melanocyte rescue at 48 hpf. At 2 months of age, adult fish that showed a nevus ≥4 mm^2^ were treated with doxycycline, so that the expression of the pigmentation modulator was induced. In the following months, induced fish were used to study the incidence, features and drug sensitivity of the melanoma tumors that form. As shown in Fig. 2C, we created miniCoopR-I-sponge-204/SCR and miniCoopR-I-pre-miR-204/SCR vectors and we used them first to test the stringency and correct functioning of our inducible system (Fig. 3), then to provide a proof of principle of its use as a tool to study the impact of pigmentation modulators on melanomagenesis (Fig. 4).

### Characterization of miniCoopR-I vectors for the inducible inhibition and overexpression of *miR-204*

Leakiness is the main limitation of inducible systems, as it compromises the controlled expression of the gene of interest. To ensure that our system is not leaky, we injected miniCoopR-I-sponge-204/SCR or miniCoopR-I-pre-miR-204/SCR vectors in 1-cell embryos of the *Tg(mitfa:BRAFV600E);p53−/−;mitfa−/−* line and 1 week later we calculated the percentage of non-induced embryos showing melanocyte rescue. Contrary to what was obtained with miniCoopR vectors (Fig. 1D and G), no change in rescue percentages was observed (Fig. 3A and E). In turn, these results suggest that miniCoopR-I vectors cannot drive *miR-204* inhibition/overexpression unless dox is added to fish water.

Next, we tested whether *miR-204* inhibition and overexpression are indeed induced by dox, at the desired stage of fish life. To do so, we used embryos injected at the 1-cell stage, selected for melanocyte rescue at 48 hpf and finally treated or not with 10 uM dox for 2 days. As far as miniCoopR-I-sponge-SCR and miniCoopR-I-sponge-204 vectors are concerned, the analyses performed by qRT-PCR (Fig. 3B), ISH (Fig. 3C) and fluorescence microscopy (Fig. 3D) indicated that, as expected, dox treatment causes an induction in *eGFP*-reporter levels in embryos injected with both vectors. In the case of miniCoopR-I-sponge-204 vector, dox treatment also causes a decrease in *miR-204* and *tyr* levels (Fig. 3B). Conversely, the analyses performed by qRT-PCR (Fig. 3F), ISH (Fig. 3G) and fluorescence microscopy (Fig. 3H) indicated that dox treatment causes the expected induction of *eGFP* reporter levels in embryos injected with both miniCoopR-I-pre-miR-SCR and miniCoopR-I-pre-miR-204 vectors. In the case of miniCoopR-I-pre-miR-204 vector, dox treatment causes an increase in *pre-miR-204* and *tyr* levels as well (Fig. 3F,G). Analogous results were obtained when the vectors were tested at later time points (9 dpf embryos after 1 week of dox treatment, Fig. S3A,B; 1-month-old juveniles after 1 week of dox treatment, Fig. S3C,D).

All together, these data attest that miniCoopR-I vectors are not leaky and display a correct functioning, therefore they can be used to turn on the inhibition or overexpression of *miR-204* at the desired time point.

### *miR-204* overexpression by miniCoopR-I increases melanoma incidence

Since our goal is to determine how *miR-204* inhibition and overexpression affect BRAFV600E-induced melanomagenesis in adult fish, we devised a schedule for long-term dox treatment. Such schedule is based on dox pulses: 10 uM dox is added to fish water for 3 days, then dox-containing water is replaced with fresh water and 12 days later a new 3-day-long pulse of dox is started (Fig. 4A). This schedule is sufficient to ensure the induction (Fig. 4B) and the constant expression (data not shown) of the transgene. Furthermore, it minimizes the number of days that fish need to be kept away from direct light (dox is light sensitive).

We started dox treatment on 2-month-old fish that had been injected with miniCoopR-I-sponge-SCR/204 or miniCoopR-I-pre-miR-SCR/204 and that presented a nevus ≥4 mm^2^. In compliance with the 3R principles, non-induced fish were not included in our analyses, so that the overall number of experimental animals was minimized. Induced fish were observed once a week, in order to monitor the malignant transformation of nevi and to score the appearance of melanoma tumors. The tumors that formed (see Fig. 4C for a representative example) were characterized, as expected, by uniform expression of BRAFV600E protein (Fig. 4D). Furthermore, those that formed in fish injected with sponge-204 or pre-miR-204 showed lower or higher levels of mature *miR-204* compared to SCR controls (Fig. 4E), a result that confirms the correct functioning of miniCoopR-I vectors in adult fish. Finally, melanoma-free survival curves indicated that sponge-mediated inhibition of *miR-204* has no effect on melanoma incidence and penetrance (Fig. 4F), suggesting that a stronger decrease in *miR-204* levels is required in order to appreciate a biological effect. On the contrary, *miR-204* overexpression does increase both (Fig. 4G). Therefore, we can conclude that the microRNA favors BRAFV600E-induced melanomagenesis in the *p53*-null background.

Taken together, the data presented indicate that *miR-204* promotes the differentiation of the melanocytic lineage during embryonic development, as well as BRAFV600E-driven melanomagenesis in adult fish. They also indicate that the tissue-specific and inducible miniCoopR-I vector that we developed and characterized allows to precisely manipulate the expression levels of the gene of interest both in time and space. Therefore, it is particularly useful for the study of genes that, like *miR-204*, are pleiotropic and exert different functions in different phases of fish life. An additional advantage of vectors like miniCoopR-I is that because they are based on Gateway technology, they are modular in nature, hence very versatile. They can be adapted to virtually any experimental need, by choosing the most suitable promoter (for total body or tissue-specific expression); gene of interest (non-coding, but also coding. Of note, besides sponge-204 or pre-miR-204, the vectors here described also express the coding eGFP mRNA, for a total transcript length of ∼1 kb); mechanism and degree of gene modulation [overexpression (Jung et al., 2019); downregulation using RNA interference (Giacomotto et al., 2015); knock in (Prykhozhij and Berman, 2018) or knock out (Kaufman et al., 2016; Ablain et al., 2018; Jung et al., 2019) using CRISPR/Cas9 technology].

## MATERIALS AND METHODS

### RNA extraction, DNAse treatment and retrotranscription

RNA was extracted using QIAzol (Qiagen), following the manufacturer's instructions. RNA was subsequently quantified using Nanodrop Lite (Thermo Scientific).

To analyze mRNA expression, 1ug of total RNA was treated with DNAse I amplification grade (Thermo-Fisher Scientific), following the manufacturer's protocol. 250 ng of RNA, treated with DNAse I, was retrotranscribed on a S1000 Thermal Cycler (Bio-Rad) using iScript cDNA Synthesis Kit (Bio-Rad). The successful retrotranscription and the absence of contaminating genomic DNA were routinely checked through a control PCR (PCR Master Mix, Thermo-Fisher Scientific) in which the exon-spanning primers for *dre-Ef1a* mRNA (see below) are used. miRNA expression analysis does not require DNAse treatment. 125 ng of RNA were retrotranscribed using miScript II RT Kit (Qiagen) and UP1 reverse primer on a S1000 Thermal Cycler (Bio-Rad).

### Real-time PCR

As previously reported (Marranci et al., 2017; Marranci et al., 2019), real-time PCR (qRT-PCR) was performed in triplicate, using 2 ul of a 1:4 dilution of cDNA, appropriate primers (0.5 uM each) and SSOADV Universal SYBR Green (Bio-Rad) in 15 ul final reaction volume on a CFX96 Real-Time System (Bio-Rad). The following amplification conditions were used: 30 s 98°C (3 s 98°C, 20 s 60°C, 10 s 72°C)×40 cycles for mRNAs and 30 s 98°C (3 s 98°C, 20 s 58°C, 10 s 72°C)×40 cycles for microRNAs. Melting curve analysis of the PCR products was added at the end of each run to assess the specificity of the reaction. Data were analyzed using CFX Manager Software (Bio-Rad). Relative expression of targets was determined using the 2^−ΔΔCt^ method and data were normalized using housekeeping genes.

For mRNA expression studies, qRT-PCR primers were designed to be exon spanning (when possible) and to produce 100-150 bp long amplicons. Two zebrafish genes were taken as housekeeping: *dre-Ef1a* and *dre-18S* (Casadei et al., 2011; McCurley and Callard, 2008).

To measure mature *miR-204* levels in zebrafish, we used as forward a primer that recognizes both *hsa-miR-204* and *hsa-miR-211* [hsa-miR-204 family fw, (Vitiello et al., 2017)] and UP1 reverse primer. The housekeeping gene was *dre-U6*.

All qRT-PCR primers are listed in Table S1.

### Plasmids

To clone miniCoopR and miniCoopR-I vectors, we took advantage of plasmids of the Tol2kit (http://tol2kit.genetics.utah.edu/index.php/Main_Page), which in turn relies on site-specific recombination-based cloning (multisite Gateway technology).

To create miniCoopR vectors (Fig. 1A), we added the Gateway™ LR Clonase™ Enzyme (Thermo-Fisher Scientific) to a mix of four plasmids: miniCoopR backbone (which contains the *mitfa* minigene and was a kind gift from Dr Yariv Houvras, Weill Cornell Medical College, New York, NY, USA); p5M-*mitfa* promoter (kind gift from Dr Charles Kaufman, Washington University School of Medicine, St. Louis, MO, USA); pME-eGFP-sponge-SCR/204 or pME-pre-miR-SCR/204; p3M-polyA (Tol2kit).

To obtain pME-eGFP-sponge-SCR/204 and pME-pre-miR-SCR/204 plasmids, the following procedure was used. Sponge-SCR contains six copies of the scrambled (non-targeting) sequence 5′-GTGTAACACGTCTATACGCCCA-3′, while sponge-204 contains six copies of a sequence fully complementary to that of *hsa-miR-204*, except for four mismatched nucleotides that create a bulge (5′-AGGCATAGGACACAAAAGGGAA-3′). The six copies were obtained using pNOT plasmid as backbone. In turn, pNOT was obtained by re-ligation of pCMV-MCS plasmid after digestion with NotI, so that the CMV promoter-driven expression cassette was removed. The sense and antisense primers containing the sequence to be repeated, flanked by XhoI restriction site on one side and SalI plus BglII restriction sites on the other side, were annealed to make a double strand fragment with sticky ends (NotI). The fragment was then phosphorylated using PNK enzyme, according to the manufacturer's instructions, and finally cloned into the pNot plasmid, previously digested with NotI. The compatible XhoI and SalI restriction sites, as well as BglII restriction site, were then appropriately used in order to duplicate the same sequence, until six copies were obtained. Finally, the six copies were digested using XhoI and SalI (see Table S2 for the sequence) and subcloned in pME-eGFP plasmid (Tol2kit), previously digested with XhoI. In this way, pME-eGFP-sponge-SCR and pME-eGFP-sponge-204 were obtained. The XhoI restriction site is located downstream of the eGFP STOP codon, therefore sponge-SCR and sponge-204 are transcribed as 3′UTR. pre-miR-SCR and *H. sapiens* pre-miR-204 were amplified by PCR from the corresponding pGIPZ vectors (Vitiello et al., 2017) and then cloned in the multicloning site of pME plasmid (Tol2kit), using XhoI and NotI restriction sites. The primers used for PCR amplification were the following: pre-miR-SCR fw: 5′-CAT**CTCGAG**AAGGTATATTGCTGTTGACA-3′ pre-miR-SCR rv: 5′-GCA**GCGGCCGC**CGAGGCAGTAGGCACTCTCG-3′ pre-miR-204 fw: 5′-CAT**CTCGAG**GACAGGGTGATGGAAAGGAG-3′ pre-miR-204 rv: 5′-GCA**GCGGCCGC**ATTTGATGATGGTGCAAT-3′.

The sequence of the amplified fragments is shown in Table S2.

To create miniCoopR-I vectors (Fig. 2C), we added the Gateway™ LR Clonase™ Enzyme to a mix of four plasmids: miniCoopR backbone; p5M-(*mitfa* promoter)(TRE-eGFP-sponge-SCR/204) or p5M-(*mitfa* promoter)(TRE-eGFP-pre-miR-SCR/204); pME-rtTA-FLAG [obtained from the Tet-On Kit (Campbell et al., 2012)]; p3M-polyA (Tol2kit).

To obtain p5M-(*mitfa* promoter)(TRE-eGFP-sponge-SCR/204) and p5M-(*mitfa* promoter)(TRE-eGFP-pre-miR-SCR/204), the following procedure was used. pNot plasmid was used to clone, one after the other, the elements that compose the TRE expression cassette: TRE promoter [amplified from the Tet-On Kit (Campbell et al., 2012)]; eGFP-sponge-SCR/204 (see above) or eGFP and then pre-miR-SCR/204 (see above); polyA (amplified from p3M-polyA, Tol2kit). Also in this case, the cloning strategy was based on XhoI/SalI and BglII restriction site. Then, the entire cassette was digested with XhoI and SalI and subcloned into p5M-*mitfa* promoter plasmid, previously digested with XhoI. The direction was opposite compared to the one of the *mitfa* promoter itself, in order to avoid transcriptional interference.

Aside from the Gateway™ LR Clonase™ Enzyme, all restriction and modification enzymes used were from New England BioLabs.

### Zebrafish transgenic lines

In this study two zebrafish lines were used: *Tg(mitfa:BRAFV600E);p53-/-;mitfa-/-* (kind gift from Dr. Yariv Houvras, Weill Cornell Medical College, New York, USA) and *p53-/-;mitfa-/-.* Animals were raised and maintained under standard laboratory conditions in a zebrafish housing system (Tecniplast).

The zebrafish facility has been authorized by the Italian Ministry of Health (authorization number: 297/2012-A, issued on 21 December 2012). All the experimental procedures were carried out in accordance with the Italian guidelines and all experimental protocols were approved by the Italian Ministry of Health (authorization number: 1222/2015-PR).

### Microinjections

MiniCoopR-based vectors (20–30 pg) and Tol2 transposase mRNA (20–30 pg) were microinjected into 1-cell *Tg(mitfa:BRAFV600E);p53−/−;mitfa−/−* or *p53−/−;mitfa−/−* embryos using a microinjector (Tritech Research). The embryos to be injected with the different vectors were chosen randomly. A 10% mortality was observed, on average. The investigators were not blinded to group allocation during injections, nor to rescue and tumor scoring.

### Dox treatment

Doxycycline hyclate (Sigma-Aldrich) was resuspended in 100% ethanol at 10 mg/ml for storage and diluted with water to a working concentration of 10 uM. Embryos were treated adding doxycycline directly in water for 2–7 days.

Juveniles were treated adding dox directly in water for 7 days. Adult fish were subjected to a dox-pulse treatment, following the schedule described in Fig. 4A. Due to light sensitivity of the drug, embryos, juveniles and adult fish were protected from light during doxycycline treatment.

### Whole-mount ISH and image processing

Whole-mount ISH was conducted as described in (Thisse and Thisse, 2008). PCR products to be used as templates were generated by PCR from appropriate plasmids, using primer pairs containing the sequence of T3 promoter (Table S3) and Phusion Flash High-Fidelity PCR Master Mix (Thermo-Fisher Scientific). The PCR products were then purified using QIAquick PCR purification kit (Qiagen). Finally, the RNA probes for ISH were generated using the PCR products as template, DIG RNA Labelling Mix 10X (Roche) and T3 polymerase (Thermo-Fisher Scientific).

ISH images were acquired using Leica M80 strereomicroscope equipped with a Nikon DS-Fi1 camera. They were then analyzed with NIS-Elements software (Nikon).

The intensity of purple signal was measured as area under the curve, using ImageJ software (http://rsb.info.nih.gov).

### Fluorescence microscopy and image processing

To acquire images, embryos and adult zebrafish were anesthetized with 0.4% Tricaine, then images were acquired with a Leica TCS SP8 Confocal Laser Scanning Microscope (CLSM) and a Leica M205FA stereomicroscope, respectively. All images were analyzed with LasX Leica Application Suite (LAS)×software.

As far as embryos are concerned, a HC PL APO CS 10x/0.40 dry objective with a 0.75 zoom factor was used to frame the entire animal. Then, tile scan plus zeta stack functions were chosen to scan the whole sample in the xyz dimension. Finally, the unidirectional scan, a 400 Hz scan speed and a sequential setting between stack were used to improve the definition of the final images, which were recorded after the incidence of the laser Argon (green signal in figure) and transmitted light (contrast methods).

### Light microscopy and image processing

Adult fish were euthanized by exposure to excess of tricaine. They were then fixed in 4% paraformaldehyde (PFA) for 24 h at +4°, dehydrated through a series of graded ethanol baths and finally embedded in paraffin. Transverse paraffin-embedded tissue sections (5um) were used. Hematoxylin and Eosin (H&E) staining was carried out using standard methods. Fontana-Masson staining (Bio-Optica) was performed following manufacturer's instructions. For IHC, tissue sections were stained with mouse anti-BRAFV600E VE1 antibody (ab228461, Abcam) diluted 1:75 in EDTA antigen retrieval buffer (Vitiello et al., 2019). The reaction was visualized using immunoperoxidase technique and 3′-5′ diaminobenzidine chromogen substrate. Images were acquired at 4x magnification, using Nikon Eclipse microscope equipped with Nikon DS-Ri2 camera, and then analyzed with Nikon NIS Elements Imaging software.

### Kaplan–Meier analysis

All animals were followed for up to 20 weeks from the first dox treatment (at 2 months of age) and melanoma-free survival curves were obtained.

Once a week, tumors were scored by visual inspection. According to published guidelines (Patton et al., 2011; Iyengar et al., 2012), there is a strong correlation between histopathologic and morphologic changes. The transition from nevus to melanoma is characterized mainly by skin thickening, accompanied by outward growth. A more marked pigmentation of the thickened area can be observed as well.

No pre-specified effect size (hence sample size) was defined.

### Statistical analyses

qRT-PCR data on embryos and juveniles (Figs 1C and F, 3B and F; Figs S1A and C, S2B, S3) were analyzed using unpaired and two-tailed Student's *t*-test (https://www.graphpad.com/quickcalcs/ttest2/). The mean±s.e.m. of two independent experiments (independent injections) is reported. In each injection, at least 20 embryos were pooled for RNA extraction. qRT-PCR data on tumors excised from adult fish were analyzed using unpaired and two-tailed Student’s *t*-test (https://www.graphpad.com/quickcalcs/ttest2/). The number of tumors (fish) used in indicated in the figure (Fig. 4E). Purple signal intensities (Fig. S2d) were analyzed using unpaired and two-tailed Student's *t*-test (https://www.graphpad.com/quickcalcs/ttest2/). At least eight fish, coming from at least two independent injections, were analyzed. The mean±s.e.m. is reported.

The % of rescued embryos (Figs 1D and G, 2A and E, Fig. S1b and d) was analyzed using Fisher's exact test (https://www.socscistatistics.com/tests/fisher/default2.aspx). The mean±s.e.m. of two independent experiments (independent injections) is reported. In each injection, at least 20 embryos were scored for melanocyte rescue. Melanoma-free survival curves were analyzed using log-rank (Mantel–Cox) test. The number of adult fish scored for melanoma appearance is indicated in the figures (Fig. 4F and G, left). These fish come from multiple injections (>3). The % of fish with melanoma (Fig. 4F and G, right) were analyzed using Fisher's exact test (https://www.socscistatistics.com/tests/fisher/default2.aspx). Values of *P*<0.05 were considered statistically significant (*P<0.05, **P<0.01, ***P<0.001, ****P<0.0001).

## Supplementary Material

Supplementary information
