# Supplementary material for: Inducible modulation of miR-204 levels in a zebrafish melanoma model
Source: Biol Open. 2020 Nov 6;9(11):bio053785. doi: 10.1242/bio.053785 (PMC7657466; doi:10.1242/bio.053785)
Supplement: Supplementary information [file biolopen-9-053785-s1.pdf]

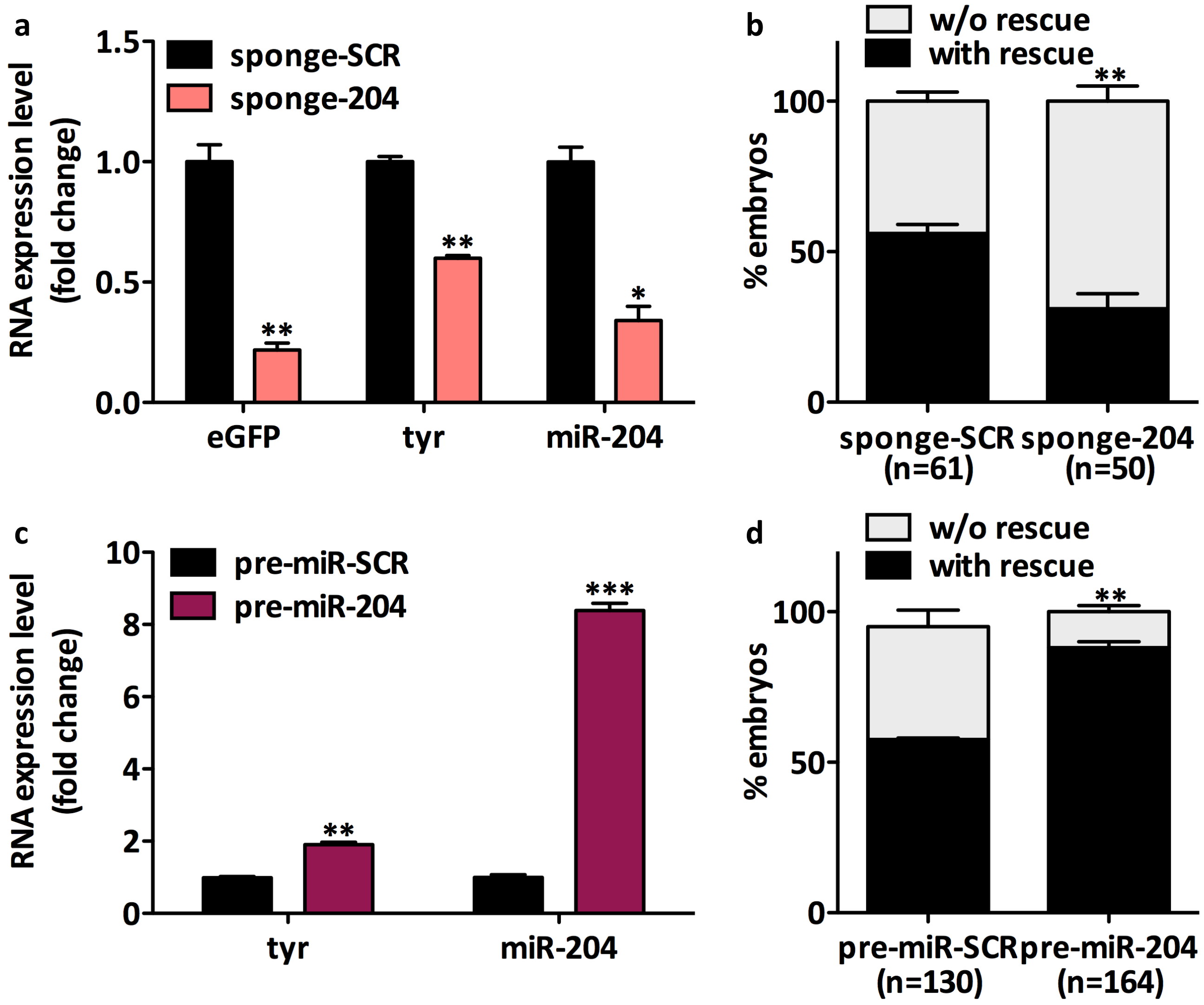

Figure S1-related to Fig.1. The constitutive modulation of miR-204 expression levels affects melanocyte content also in embryos of the *p53-/-;mitfa-/-* line.

When miR-204 levels are modulated in a genetic background that is *p53* and *mitfa* null, but *wt* for BRAF, the effects observed on melanocyte content are the same observed in the *Tg(mitfa:BRAFV600E);p53-/-;mitfa-/-* line. **(a-b)** The inhibition of miR-204 leads to lower levels of *tyr* mRNA and a lower percentage of embryos with melanocyte rescue at 7 dpf. **(c-d)** Opposite effects on all parameters are observed when miR-204 is overexpressed.

Statistically significant differences are indicated with asterisks: *p < 0.05, **p < 0.01, ***p < 0.001.

Biology Open • Supplementary information

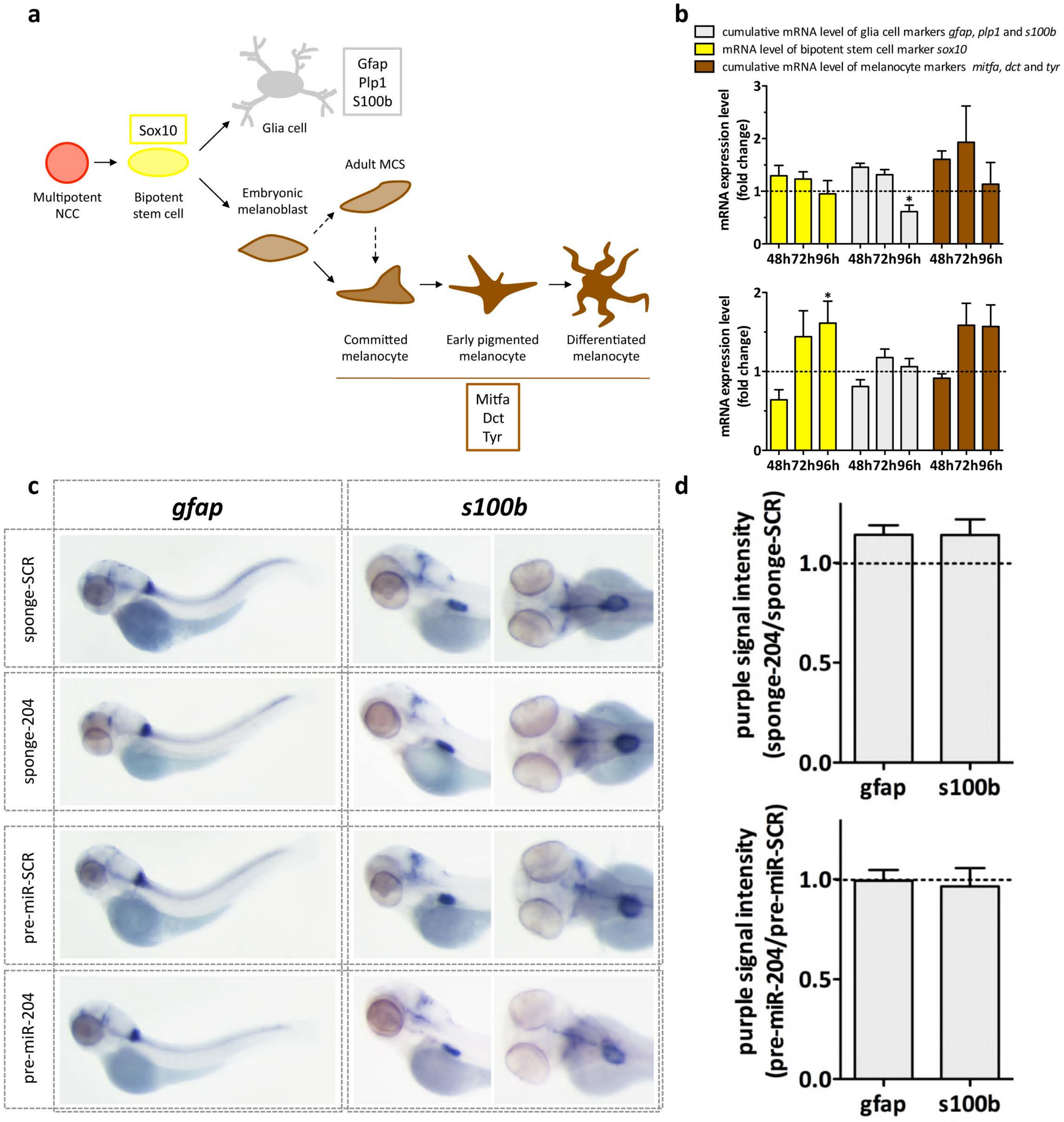

Figure S2-related to Fig. 1. Effect exerted by miR-204 inhibition and overexpression on glia and melanocyte lineages in the *Tg(mitfa:BRAFV600E);p53-/-;mitfa-/-* line.

(a) Schematic representation of the differentiation of multipotent neural crest cells (NCCs, red) during zebrafish embryonic development. Multipotent NCCs become bipotent stem cells (yellow) that are able to give origin to both glia cells (grey) and melanocytes (brown). The melanocyte specification pathway is composed, in this order, by: embryonic melanoblasts, committed melanocytes, early pigmented melanocytes and terminally differentiated melanocytes. Embryonic melanoblasts give rise to adult melanocyte stem cells (MCSs) as well. The main markers associated with each lineage are as follows. Bipotent stem cells: Sox10 (yellow). Glia cells: Gfap, Plp1 and S100b (grey). Melanocytes: Mitfa, Dct, Tyr (brown). **(b)** mRNA expression levels of the lineage markers listed and color-coded in **a**, as measured at the indicated hours post-fertilization on embryos of the *Tg(mitfa:BRAFV600E);p53-/-;mitfa-/-* line that had been injected with miniCoopR-sponge-SCR/204 (*upper*) and miniCoopR-pre-miR-SCR/204 (*lower*) at 1-cell stage. To obtain cumulative levels of glia cell and melanocyte markers (which we considered more informative about the trend of glial cells vs melanocytes compared to the levels of single markers), we summed the values obtained from embryos injected with sponge-204 or pre-miR-204 and then divided them by the sum of the values obtained from the injection of sponge-SCR and pre-miR-SCR, respectively. **(c-d)** ISH detection of *gfap* and *s100b* in 72hpf embryos of the *Tg(mitfa:BRAFV600E);p53-/-;mitfa-/* line, injected with the indicated miniCoopR vectors at 1-cell stage. In **c**, representative images are shown. In **d**, the intensity of the purple signal was measured and the area under the curve was calculated using ImageJ software.

Statistically significant differences are indicated with asterisks: $^*p < 0.05$.

Biology Open • Supplementary information

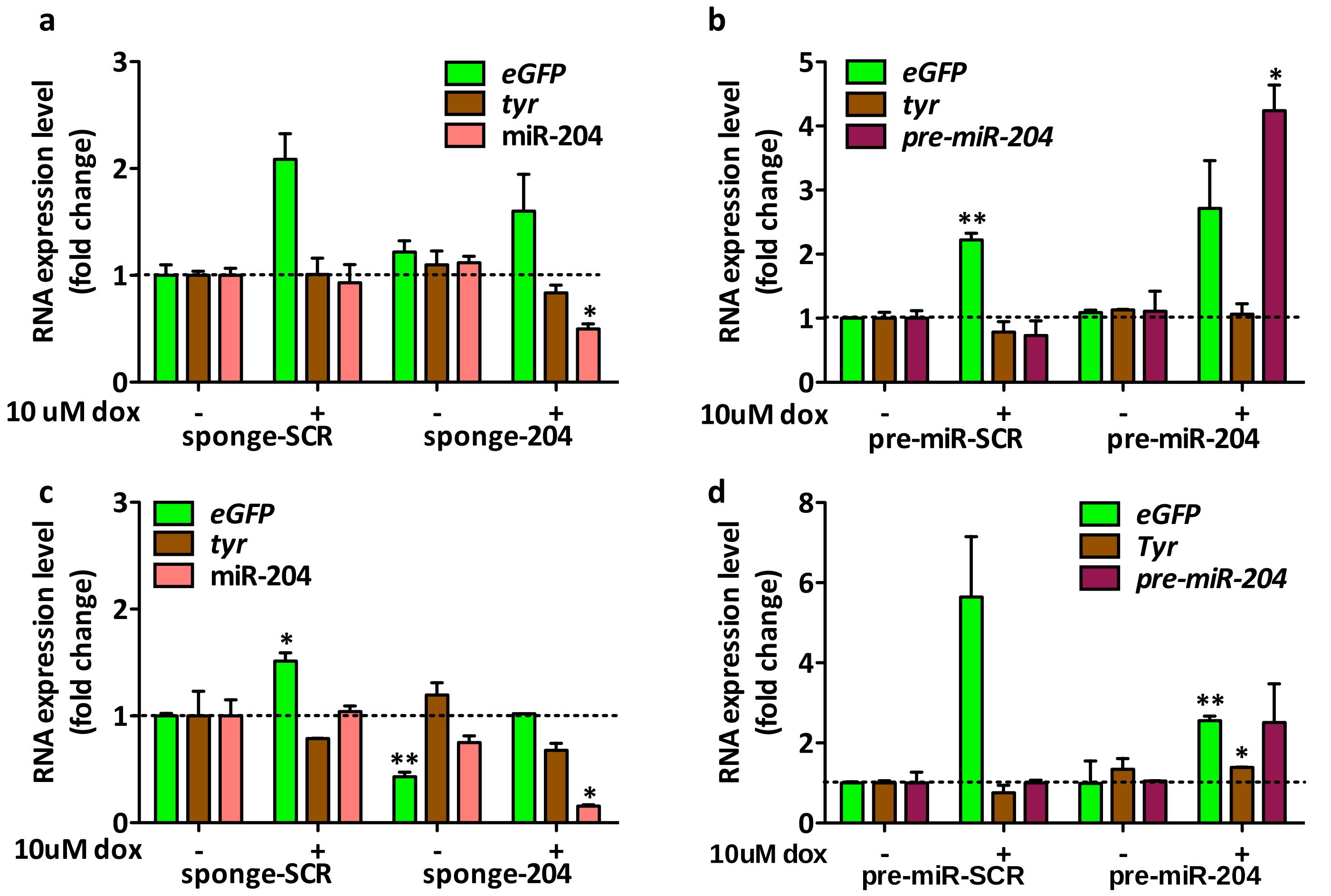

Figure S3-related to Fig.3. Inducible modulation of miR-204 expression levels in embryos of the *Tg(mitfa:BRAFV600E);p53-/-;mitfa-/-* at later stages of development.

(a-b) The levels of *eGFP*, *tyr*, mature miR-204 and *pre-miR-204* were measured on embryos injected with miniCoopR-I-sponge-SCR or miniCoopR-I-sponge-miR-204 **(a)** and miniCoopR-I-pre-miR-SCR or miniCoopR-I-pre-miR-204 **(b)**, after a longer period of treatment with 10uM dox (7 days). **(c-d)** The system was tested also in juvenile fish (1 month-old), after 1 week of dox treatment.
Statistically significant differences are indicated with asterisks: *p < 0.05, **p < 0.01.

Biology Open • Supplementary information

Table S1. Primers used for qRT-PCR

NAME	SEQUENCE	USE
dre-Ef1a fw	CTGGAGGCCAGCTCAAACAT	housekeeping primers for qRT-PCR
dre-Ef1a rev		
dre-18S fw	TCGCTAGTTGGCATCGTTTATG	
dre-18S rev	CGGAGGTTCGAAGACGATCA	
dre-Sox10 fw	AGGGAGGAAAATCAGGCGAG	RNA-specific primers for qRT-PCR
dre-Sox10 rev	TTCGCCAATGTCCACGTTAC	
dre-Mitfa fw	GATGCTCGAGTACAGTCACTACCA	
dre-Mitfa rev	GCTTCACCTGCTGCCTCT	
dre-Dct fw	CACCTGGCACAGATATCACCT	
dre-Dct rev	CGGCGAAGTTCTCATTACCT	
dre-Tyr fw	CGAGAGGCAGAGGTTCATCT	
dre-Tyr rev	GTGGAGCCGTTGTTCATCTG	
dre-Gfap fw	TGAGAGAGATGGAGGAACGC	
dre-Gfap rev	GGCCAGTTTGACATTGAGCA	
dre-Plp1 fw	GGTCTGGCCTCCTTCTTCTT	
dre-Plp1 rev	CGAACTGTGTGCTCCTGAAC	
dre-S100b fw	GACTTAGAGAACTGCCTGGGA	
dre-S100b rev	GCTTGGTCTTTCACTTGCTCA	
eGFP fw	ACGTAAACGGCCACAAGTTC	
eGFP rev	CGTAGGTCAGGGTGGTCAC	
hsa-pre-miR-SCR fw	TGGGCGAGAGTAAGTAGTGA	
hsa-pre-miR-204 fw	ACAGGGTGATGGAAAGGAG	
polyA rev	AACTTGTTTATTGCAGCTTATAA	
dre-U6 fw	ATGACACGCAAATCCGTGAAG	housekeeping primer for qRT-PCR
hsa-miR-204 family fw	GGGTACAGCATCTCGGTGTT	miRNA-specifc primer for qRT-PCR
UP1 rev	TGAATCGAGCACCAGTTACGC	reverse primer for retrotranscription and qRT-PCR

Table S2. Sequence of the inserts of miniCoopR and miniCoopR-I vectors

SEQUENCE of INSERTS
**sponge-SCR:**<br><br>TCGAG**GTGTAACACGTCTATACGCCCA**GTCGAG**GTGTAACACGTCTATACGCCCA**GTCGAG **GTGTAACACGTCTATACGCCCA**GTCGAG**GTGTAACACGTCTATACGCCCA**GTCGAG**GTGTA ACACGTCTATACGCCCA**GTCGAG**GTGTAACACGTCTATACGCCCA**G<br>**bold: scrambled (non-targeting) sponge sequence**<br>underlined: restriction sites
**sponge-204:**<br><br>TCGAG**AGGCATAGGA**CACA**AAAGGGAA**GTCGAG**AGGCATAGGA**CACA**AAAGGGAA**GTC GAG**AGGCATAGGA**CACA**AAAGGGAA**GTCGAG**AGGCATAGGA**CACA**AAAGGGAA**GTCG AG**AGGCATAGGA**CACA**AAAGGGAA**GTCGAG**AGGCATAGGA**CACA**AAAGGGAA**G<br>**bold: sponge sequence for hsa-miR-204**<br>**(nucleotides that do not match with the miRNA sequence are in grey)**<br>underlined: restriction sites
**pre-miR-SCR:**<br><br>AAGGTATATTGCTGTTGACAGTGAGCGATCTCGCTTGGGCGAGAGTAAGTAGTGAAGCCAC AGATGTACTTACTCTCGCCCAAGCGAGAGTGCCTACTGCCTCG
**pre-miR-204:**<br><br>GACAGGGTGATGGAAAGGAGGGTGGGGGTGGAGGCAAGCAGAGGACTTCCTGATCGCG TACCCATGGCTACAGTCTTTCTTCATGTGACTCGTGGAC**TTCCCT TTGTCATCCTATGCCT**GAGAATATATGAAGGAGGCTGGGAAGGCAAAGGGACGTTCAATTG TCATCACTGGCATCTTTTTTGATCATTGCACCATCATCAAATGC<br>**bold: mature hsa-miR-204 sequence**

Biology Open • Supplementary information

Table S3. Primers used for the PCR amplification of ISH probes

NAME	SEQUENCE
eGFP PROBE fw	CCCTGGCCCACCCTCGTGAC
eGFP T3-PROBE rev	AATTAACCCTCACTAAAGGGCCGTCCTCGATGTTGTGGCG
hsa-pre-miR-204 PROBE fw	GACAGGGTGATGGAAAGGAGGGT
hsa-pre-miR-204 T3-PROBE rev	AATTAACCCTCACTAAAGGGGCATTTGATGATGGTGCAATGAT
dre-Gfap PROBE fw	CGTGCAGCTAGAGAGGAAGA
dre-Gfap T3-PROBE rev	AATTAACCCTCACTAAAGGGATTCCAGGTCACAGGTCAGG
dre-S100b PROBE fw	GACTTAGAGAACTGCCTGGGA
dre-S100b T3-PROBE rev	AATTAACCCTCACTAAAGGGGAAATCACACTCCGAGTCGC

Biology Open • Supplementary information
